# Characterization of Organoid Cultures to Study the Effects of Pregnancy Hormones on the Epigenome and Transcriptional Output of Mammary Epithelial Cells

**DOI:** 10.1007/s10911-020-09465-0

**Published:** 2020-11-01

**Authors:** Michael F. Ciccone, Marygrace C. Trousdell, Camila O. dos Santos

**Affiliations:** Cold Spring Harbor Laboratory, Cold Spring Harbor, NY 11724 USA

**Keywords:** Mammary organoids, Pregnancy-induced development, Epigenomics

## Abstract

The use of mouse derived mammary organoids can provide a unique strategy to study mammary gland development across a normal life cycle, as well as offering insights into how malignancies form and progress. Substantial cellular and epigenomic changes are triggered in response to pregnancy hormones, a reaction that engages molecular and cellular changes that transform the mammary epithelial cells into “milk producing machines”. Such epigenomic alterations remain stable in post-involution mammary epithelial cells and control the reactivation of gene transcription in response to re-exposure to pregnancy hormones. Thus, a system that tightly controls exposure to pregnancy hormones, epigenomic alterations, and activation of transcription will allow for a better understanding of such molecular switches. Here, we describe the characterization of ex vivo cultures to mimic the response of mammary organoid cultures to pregnancy hormones and to understand gene regulation and epigenomic reprogramming on consecutive hormone exposure. Our findings suggest that this system yields similar epigenetic modifications to those reported in vivo, thus representing a suitable model to closely track epigenomic rearrangement and define unknown players of pregnancy-induced development.

**Supplementary Information:**

The online version of this article (10.1007/s10911-020-09465-0) contains supplementary material, which is available to authorized users.

## Introduction

The mammary gland is one of the few organs that undergoes multi-stage development post-birth. Although significant changes mark the pre- and post-pubescence mammary developmental stages, those associated with pregnancy have the greatest effect on cellular and tissue reorganization.

The adaptation of hormonal conditions to mimic many of the cellular and molecular events brought to the mammary epithelial cells (MECs) by pregnancy may allow for better dissection of signaling and responses that would otherwise be masked during tissue processing or untimed hormone exposure. Most importantly, such strategy would support the development of systems to understand the influence of pregnancy hormones in MECs from a variety of animal species, thus providing a suitable platform to understand pregnancy induced mammary development from an evolutionary perspective.

For the past several years, optimized three dimensional (3D) organoid cultures have provided a strong and reliable platform to dissect normal and malignant mammary development. In fact, several studies have demonstrated that inclusion of several hormone cocktails to 3D cultures induces branching morphogenesis [1–5] and developmental stage transitions, such as lactation and involution [6]. In addition, 3D organoid cultures allow for the validation of mammary focused phenotypes observed in genetically engineered mouse models [7–9] and during mammary oncogenesis [10, 11]. These findings support its relevance to define nuances of mammary development in a more tightly controlled strategy.

Several studies have reported that a pregnancy cycle (gestation, lactation and involution) induces stable modifications to the epigenome of MECs, which influence normal development and breast cancer risk [12, 13]. More specifically, post-pregnancy MECs were shown to retain an epigenetic memory from previous pregnancies, marked by stable loss of DNA methylation at specific regulatory regions, which influences the expression of milk-associated proteins in consecutive exposure to pregnancy hormones [14, 15]. More recently, this rapid increase on gene expression was also observed in organoid cultures derived from post-pregnancy mammary tissue, therefore supporting a cell autonomous regulation of such epigenetic memory [1].

Here, we demonstrate that organoid cultures also represent a suitable system to understand the molecular changes brought to MECs by pregnancy hormones. Using this approach, we provide a more in-depth picture of the dynamics of gene expression and active regulatory regions of 3D organoid cultures derived from pre- and post-pregnancy mammary tissue. Our strategy demonstrates how readily the epigenomic and transcriptomic changes are in response to pregnancy hormones, and how such changes are enhanced in cells re-exposed to pregnancy hormones. Moreover, we utilized our system to validate the role of a known mammary regulatory factor, EZH2, on organoid development, a strategy that yielded hypothesis for its role in controlling gene expression in response to re-exposure to pregnancy hormones. Further utilization of such robust and highly controlled 3D culturing system will undoubtedly improve our knowledge of activation or repression of processes that influence oncogenesis in a pregnancy dependent manner. 

## Results

### Pregnancy Hormones Induce Robust Upregulation of Casein 2 (Csn2) in Post-Pregnancy Mammary Organoid Cultures

**Fig. 1:**
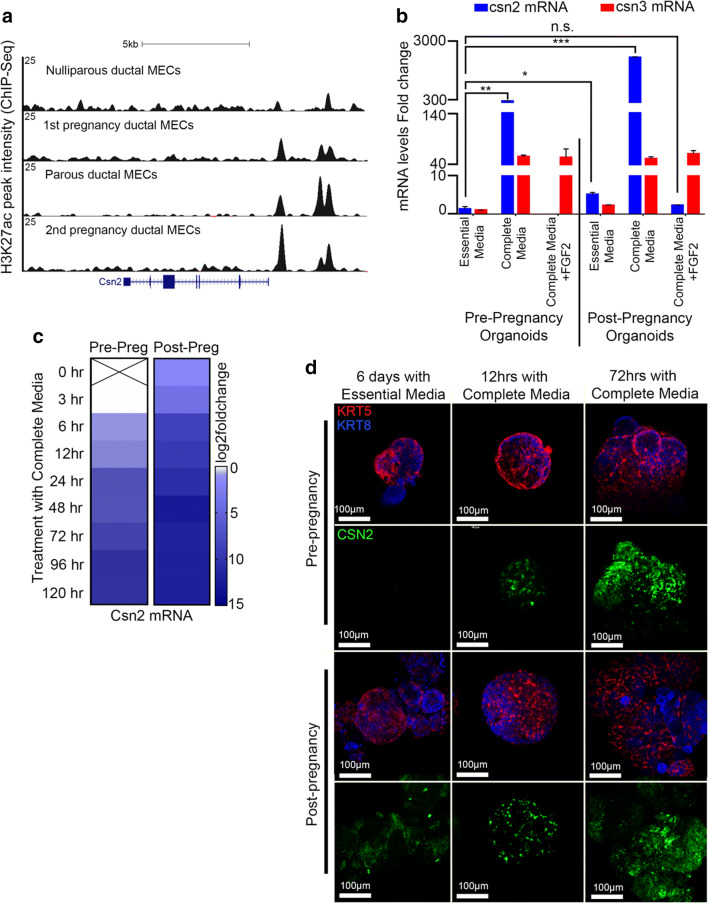
**Pregnancy hormones induce robust upregulation of Casein 2 (Csn2) in post-pregnancy mammary organoid cultures.** (**a**) Genome browser tracks showing distribution of H3K27Ac peaks at the Csn2 locus in luminal ductal MECs harvested from pre- and post-pregnancy mice at distinct pregnancy cycles. (**b**) mRNA levels (qPCR) of casein genes, *Csn2* and *Csn3*, in pre- and post-pregnancy mammary organoids grown with essential medium, complete medium (supplemented with pregnancy hormones) or complete medium with FGF2 *n* = 2 biological replicates, with 3 technical replicates each. ***p = 0.0002, **p = 0.002, *p = 0.01 (**c**) Csn2 mRNA levels (2^deltadeltaCT) in pre-pregnancy mammary organoids grown with complete medium. Normalized to pre- pregnancy organoids treated with complete medium for 3 h. (**d**) Immunofluorescence (IF) imaging of whole mounted pre- and post-pregnancy mammary organoids before or after complete medium exposure for 12 h and 72 h. KRT8 (blue), KRT5 (red) and CSN2 (green). Scale: 100 μm. For analyses, error bars indicate standard error of the mean (SEM) across samples of same experimental group. *p* values were defined using Welch’s t-test

Our previous work identified pregnancy induced changes in the epigenome of MECs [14]. More recently, we utilized chromatin immune precipitation sequencing (ChIP-seq) and demonstrated a substantial expansion of the active regulatory landscape of post-pregnancy MECs [1]. Amongst the alterations brought by a full pregnancy cycle, we found two regions upstream of the Csn2 genomic locus, a milk associated protein, to stably gain H3K27ac marks after pregnancy (Fig. 1a). Such pregnancy-induced alterations were associated with a 10-fold increase in *Csn2* mRNA levels and protein levels in flow cytometry isolated, post-pregnancy luminal cells and organoid cultures treated with pregnancy hormones [1]. Based on these findings, we predict that Csn2 levels can act as a reporter marker to track gene reactivation of pregnancy-induced epigenome.

In order to investigate the conditions that support increased gene expression in response to pregnancy hormones, we derived organoid cultures utilizing pre- and post-pregnancy mammary tissue and measured *Csn2* and Casein 3 (*Csn3*), an additional milk associated protein, mRNA levels from cultures grown at several conditions (Supplementary Fig.S1). Our results confirmed that *Csn2* mRNA levels were ~ 9-fold higher in post-pregnancy organoids grown with pregnancy hormones, than in pre-pregnancy organoids grown under the same conditions (Fig. 1b). Interestingly, *Csn2* mRNA levels failed to increase in pre- and post-pregnancy organoids grown with pregnancy hormones and Fibroblast Growth Factor 2 (FGF2), suggesting that the proliferation and self-renewal programs known to be regulated by FGF2 [16, 17] may have interfere with the differentiation and specialization process driven by pregnancy hormones in mammary organoids. Moreover, *Csn3* mRNA levels were equally induced in pre- and post-pregnancy organoids grown with complete medium (~47- and ~ 48-fold, respectively), independently of FGF2 presence, thus suggesting that its expression is regulated by pregnancy hormones independent of a previous exposure to pregnancy hormones.

We next set out to more precisely define the dynamics of *Csn2* mRNA levels in pre- and post-pregnancy organoids using a time-course response to pregnancy hormones. Our results suggested that *Csn2* mRNA level differences peaked after 6 hours (h) of pregnancy hormones treatment, with post-pregnancy organoids expressing ~20x fold higher mRNA levels than pre-pregnancy ones (Fig. 1c). Such differences in mRNA levels were normalized after 96 h of pregnancy hormone treatment, a time point where pre- and post-pregnancy organoids expressed similar levels of *Csn2* mRNA. Accordingly, CSN2 protein levels peaked higher in post-pregnancy mammary organoids in response to pregnancy hormone treatment, a result that agrees with previous observations [1] (Fig. 1d). Collectively, these results confirm that rapid gene expression activation in response to pregnancy hormones are maintained in organoid cultures derived from post-pregnancy MECs, and suggests the utilization of Csn2 levels as a reporter marker to track pregnancy-induced epigenetic gene expression reactivation.

### Pregnancy Hormones Drive Changes to the Active Regulatory Landscape of Pre-Pregnancy Mammary Organoid Cultures

**Fig. 2:**
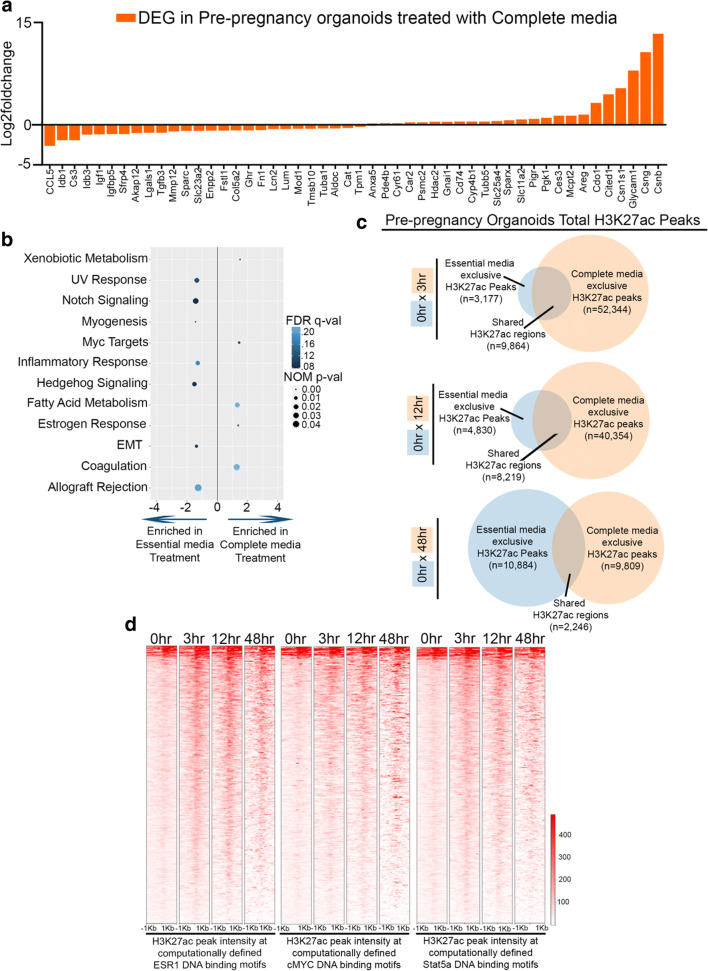
**Pregnancy hormones drive changes to the active regulatory landscape of pre-pregnancy mammary organoid cultures.** (**a**) RNAseq quantification of parity gene signature in pre-pregnancy organoids treated with complete medium for 9 days. n = 2 biological replicates. (**b**) GSEA of gene networks differentially expressed in pre-pregnancy organoids treated with complete medium for 9 days. (**c**) Venn diagrams comparing H3K27ac peaks of pre-pregnant mammary organoid cultures before complete medium treatment (0 h) and after 3 h, 12 h and 48 h treatment with complete medium. (**d**) Density plot showing H3K27ac levels at computationally defined DNA binding motifs recognized by the transcription factors ESRa, MYC and STAT5 in pre-pregnancy mammary organoids before complete medium treatment (0 h) and after 3 h, 12 h, 48 h treatment with complete medium

We next decided to fully characterize the alteration to the transcription output and epigenome of pre-pregnancy mammary organoids in response to pregnancy hormones. Thus, we utilized a previously published RNA-seq data set that compared the transcriptome of whole mammary tissue from nulliparous and parous rats and mice with whole mouse mammary gland [18], to define whether a signature of parity was present in pre-pregnancy organoid cultures grown with pregnancy hormones (Complete medium, 9 days of treatment). We found that 31% of such parity signature was present in pre-pregnancy organoid cultures grown with pregnancy hormones, thus suggesting that a signature of parity was established during in vitro exposure to pregnancy hormones, (Fig. 2a and Supplementary Table S1). Gene Set Enrichment Analysis (GSEA) demonstrated that signatures of active proliferation [19, 20] (Myc targets), milk synthesis [21] (fatty acid metabolism) and estrogen response [22] were enriched in pre-pregnancy organoid cultures grown with pregnancy hormones, thus supporting their pregnancy- like development (Fig. 2b). Interestingly, signatures associated with immune response and stem-like state developmental processes, known to be suppressed during pregnancy [23–25], were downregulated in pre-pregnancy organoid cultures grown with pregnancy hormones, thus supporting that our system incorporates a complete array of transcriptional modifications associated with mammary pregnancy development.

Given that treatment with pregnancy hormones induced pregnancy associated transcriptional changes to organoid cultures, we next investigated whether changes to the active regulatory landscape were also established by such treatment. In doing so, we utilized Cleavage under targets and release using nuclease (Cut&Run), a strategy that allows for immunoprecipitation of native chromatin [26] to profile the active histone mark H3K27ac in pre-pregnancy mammary organoids treated with pregnancy hormones for 3 h, 12 h and 48 h. Total H3K27ac peak analysis demonstrated a ~ 40-fold expansion of the number of H3K27ac peaks in mammary organoids treated with pregnancy hormones for the first 3 h and 12 h (Fig. 2c). Such expansion likely activated putative enhancer regions, given that analyses of total H3K27ac peaks genomic distributions indicated a ~ 5-fold and ~ 4-fold increase, respectively, in the number of peaks mapping to intergenic and genic regions, supporting the notion that pregnancy induces changes to the enhancer landscape of MECs (Supplementary Fig. S2A and S2B). Our analyses also demonstrated a 4 to 3-fold decrease on the number of H3K27ac peaks shared between organoids with no treatment (0 h) to those grown with pregnancy hormones for 48 h, in comparison to those shared between 0 h and 3 h, and 0 h and 12 h, respectively, suggesting a greater divergence of active regulatory landscape after prolonged exposure to hormones (Fig. 2c, bottom panel).

Gene Ontology (GO) analyses suggested that H3K27ac peaks exclusive to organoids after hormone treatment for 3 h were associated with genes that play a role in adherent junction organization and response to epidermal growth factor, while peaks exclusive to organoids treated with hormones for 12 h were enriched for genes associated with negative regulation of notch signaling and positive regulation of actin filament bundle assembly, all processes previously linked with pregnancy development [27–30] (Supplementary Fig. S2C). Amongst the networks predicted to be associated with H3K27ac peaks exclusive to organoids after hormone treatment for 48 h, we identified terms associated with unfolded protein response, protein catabolism and endoplasmic reticulum response, all pathways previously described to be essential for the post-pregnancy involution process of mammary glands [31–35] (Supplementary Fig. S2C, bottom panel). In fact, it was recently demonstrated that pregnancy-induced mammary involution can be recapitulated in vitro [6], thus supporting an involution-like state to organoid cultures treated for 48 h with pregnancy hormones, an observation that we are now supplementing with molecular signatures. Moreover, we also observed that a fraction of all pregnancy hormone exclusive peaks were shared across all 3 timepoints, suggesting sustainability of epigenetic changes established by pregnancy signals throughout the treatment time-course (Supplementary Fig. S2D).

We next set out to define whether specific Transcription Factor (TF) DNA motifs were enriched in regions that gained H3K27ac peaks after hormone treatment (complete medium exclusive peaks). Our analyses identified gain of H3K27ac peaks at DNA motifs predicted to be occupied by the TFs Interferon responsive factor 1 (IRF1) and Forkhead box protein C2 (FOXC2), previously described to enhance proliferation and suppress apoptosis of MECs [36, 37], in organoid cultures treated with pregnancy hormones for 3 h and 12 h, suggesting that signals of immediate response to pregnancy hormones remained active for many hours (Supplementary Fig. S2E). Conversely, our analyses identified DNA motifs for a different set of TFs in organoid cultures treated with pregnancy hormones for 48 h, including the Specificity protein 3 (SP3), which has been implicated in regulating gap junction formation during pregnancy and lactation [38]. These results further suggest that prolonged hormone exposure in vitro drives molecular programs that could be demarking a differential developmental stage, as suggested by the analyses of terms associated with H3K27ac peaks exclusive to organoid cultures treated with pregnancy hormones for 48 h (Fig. 2c, bottom panel).

Surprisingly, this unbiased TF DNA motif analyses did not indicate enrichment of classical regulators of mammary pregnancy-induced development such as Estrogen receptor α (ERα) [39, 40], cellular Myelocytomatosis (cMYC) [20] and Signal transducer and activator of transcription 5 (STAT5) [41, 42], at H3K27ac peaks exclusive to organoid treated with pregnancy hormones, suggesting that perhaps these TFs could be associating with chromatin, even in the absence of pregnancy hormones. To address this point, we analyzed H3K27ac peaks intensity of computationally defined DNA motifs recognized by ERα (EREs), cMYC (eBOX) and STAT5 (GAS), and found increased peak intensity at these DNA motifs in response to treatment with pregnancy hormones, suggesting that some of these regions may be occupied by such TFs during organoid growth and expansion, and further gain of H3K27ac levels in response to pregnancy hormones which may represent increased TF binding and enhanced gene expression activation (Fig. 2d).

### Altered Epigenomic Landscape Drives Post-Pregnancy Mammary Organoids Response to Re-Exposure to Pregnancy Hormones

**Fig. 3:**
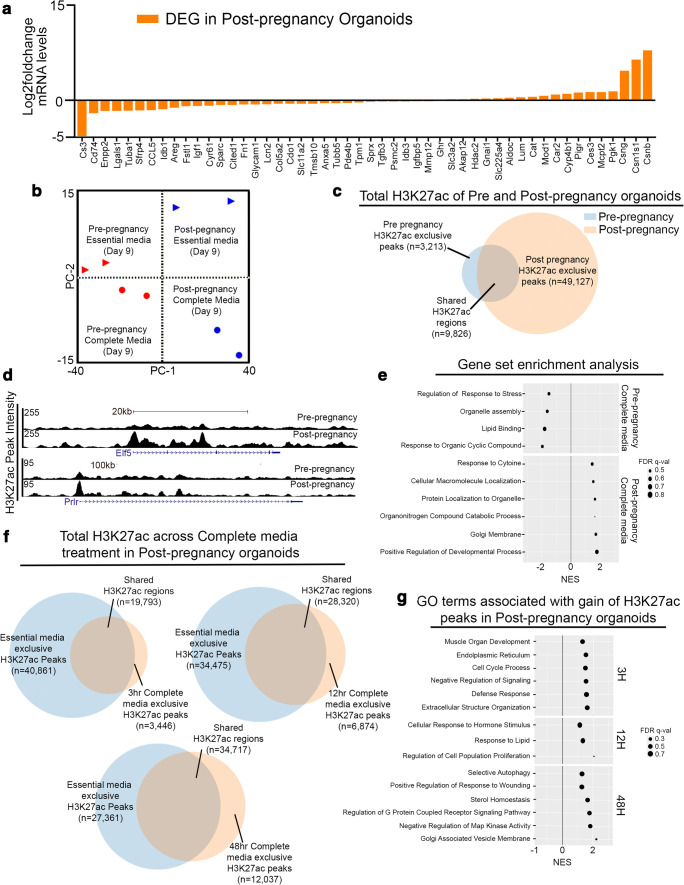
**Altered epigenomic landscape drives post-pregnancy mammary organoids response to re-exposure to pregnancy hormones.** (**a**) RNAseq quantification of parity gene signature in post-pregnancy mammary organoids grown with essential media (no pregnancy hormones) for 9 days. n = 2 biological replicates. (**B**) Principal component analyses of gene expression datasets from pre- and post-pregnancy organoids treated with and without complete medium for 9 days. (**c**) Venn diagram comparing total H3K27ac peaks of untreated pre-pregnant mammary organoids and post-pregnancy mammary organoids. (**d**) Genome browser tracks showing distribution of H3K27ac peaks in untreated pre- and post-pregnancy mammary organoid cultures for the Elf5 and Prlr loci. (**e**) S-plot showing untreated post-pregnancy organoid exclusive H3K27ac peaks intersected with gene expression from untreated post-pregnancy organoids day 9. (**f**) Venn diagrams comparing H3K27ac peaks from post-pregnant mammary organoid cultures before complete medium treatment (0 h) and after 3 h, 12 h and 48 h of treatment with complete medium (**g**) GSEA of gene networks exclusive to post-pregnancy mammary organoids treated with complete medium for 3 h, 12 h and 48 h

Having established the effects of pregnancy hormone treatment on gene expression and on the epigenome of pre-pregnancy mammary organoids, we next investigated the dynamics of epigenomic remodeling in post-pregnancy organoids cultures in response to pregnancy hormones. We confirmed that post-pregnancy mammary organoids grown with essential medium (no hormones) retained a parity gene signature with 36% of the genes matching the originally described gene expression analyses, suggesting that mammary organoid culturing does not erase a transcription state established by a previous pregnancy (Fig. 3a and Supplementary Table S2). Moreover, principal component analysis of RNAseq datasets derived from untreated and hormone-treated pre- and post-pregnancy organoid cultures demonstrate grouping of transcriptomes based on treatment with pregnancy hormones (PC-1) and parity (PC-2), further suggesting that the post-pregnancy organoids, independent of complete medium treatment, display a unique gene expression profile in organoid cultures. This is further supported by the clustering of pre- and post-pregnancy organoid cultures with complete medium, which shows their expression profiles are different from their untreated counterparts, but similar to each other (Fig. 3b).

In order to define whether the unique transcriptome of post-pregnancy mammary organoids have an epigenetic basis, we employed active histone mark H3K27ac Cut&Run. Initial comparisons of total H3K27ac peaks in untreated organoids demonstrated a ~ 15-fold expansion of the active epigenomic landscape in post-pregnancy mammary organoids, a substantial alteration to the epigenomic landscape brought by pregnancy that was previously reported [1, 14] and that was retained after organoid culturing (Fig. 3c). Such expansion to the active epigenomic landscape was mostly observed on intergenic and genic regions, given the 5-fold and 4.6-fold increase of H3K27ac peaks at these regions in post-pregnancy organoids, in comparison to pre-pregnancy organoids (Supplementary Fig.S2 A and Supplementary Fig. S3A). Furthermore, we identified 14,441 genes that were associated with H3K27ac peaks exclusive to untreated post-pregnancy organoids, which included genomic regions associated with E74-like factor 5 (Elf5) and Prolactin receptor (Prlr) genes, known players of mammary gland development and lactation [43, 44] (Fig. 3d). We utilized this list of genes to identify the dynamics of gene expression regulation using RNAseq datasets from pre- and post-pregnancy organoid treated with pregnancy hormones.

In total, we identified 350 Differentially Expressed Genes (DEGs, fold change greater than 4) across pre- and post-pregnancy mammary organoids treated with pregnancy hormones, that associated with the H3K27ac peaks exclusive to untreated post-pregnancy organoids, thus supporting the notion that many of the genes with expression influenced by pregnancy hormones are epigenetically altered in post-pregnancy MECs (Supplementary Table S3). Further gene set enrichment analyses of H3K27ac-associated DEGs demonstrated enrichment of pathways associated with Golgi and Endoplasmic reticulum processes (post-pregnancy organoids treated with pregnancy hormones), and cytoplasmic transport and lipid-binding (pre-pregnancy organoids treated with pregnancy hormones), further suggesting that an array of regulatory networks that demark milk production-like processes and those associated with involution are represented in the epigenome of post-pregnancy MECs (Fig. 3e).

We next set out to define changes to post-pregnancy epigenomic landscape in response to re-exposure to pregnancy hormones. Genomic distributions analyses of H3K27ac peaks across untreated (0 h) and hormone treated (3 h, 12 h and 48 h) post-pregnancy mammary organoids indicated a minor increase on the percentage of intergenic regions across all treated timepoints (3 h = 11.8%, 12 h = 15.0% and 48 h = 18.9%) compared to untreated (0 h = 19.3%) suggesting that pregnancy hormones do not dramatically modify the post-pregnancy landscape (Supplementary Fig. S3A). Interestingly, comparison of total H3K27ac peaks from untreated post-pregnancy mammary organoids with peaks present in pre-pregnancy organoids treated for 3 h with pregnancy hormones, demonstrated an overlap of 55% of all peaks, thus supporting that the epigenome of post-pregnancy mammary organoids were established by signals present during pregnancy, and that a substantial fraction of these regions were associated with a response to pregnancy hormones (Supplementary Fig. S3B).

Further analyses of total H3K27ac peaks demonstrated an overall 1.7-fold increase on the percentage of shared peaks and a 3.5-fold increase on the percentage of hormone-treated exclusive peaks across the pregnancy hormone treatment time points, further supporting that hormone treatment did not dramatically modify the post-pregnancy landscape (Fig. 3f). Nonetheless, regions that gained H3K27ac peaks in post-pregnancy organoids treated with hormones were associated with a series of genes involved with branching morphogenesis (3 h), cell contraction and calcium transport (12 h), and endoplasmic reticulum process (48 h). These results suggest a progression from tissue expansion (3 h), lactation-like (12 h) and involution-like (48 h) stages of development (Supplementary Fig. S3C). In fact, IF analyses of organoids grown with pregnancy hormones illustrates a differential morphology of myoepithelial cells (Cytokeratin 5, KRT5+ cells, red) specifically in post-pregnancy organoid cultures, a phenotype that may indicate contractibility of such cells [45, 46] and further support their advanced developmental stage in response to pregnancy hormones (Supplementary Fig. S3D).

Surprisingly, our analyses showed that ~50% of all H3K27ac peaks in post-pregnancy organoids detected in untreated cultures failed to gain active histone marks in cultures treated with pregnancy hormones, suggesting that a fraction of the pregnancy-induced epigenome does not get immediately altered in response to pregnancy hormones (Fig. 3f). Analyses of the genes associated with these regions indicated an enrichment for pathways controlling DNA damage, response to oxidative stress and immune communication (Supplementary Fig. S3E). These results suggest that a series of tissue homeostasis pathways are constantly activated in post-pregnancy MECs, and their suppression in response to pregnancy hormones highlight additional mechanisms that may play a role on enhancing post-pregnancy MECs development in consecutive exposure to pregnancy hormones.

With the notion that post-pregnancy mammary organoids display many of the H3K27ac peak gain present in pre-pregnancy organoids treated with hormones (Supplementary Fig. S3B), we set out to define differential H3K27ac levels across hormone treatment in pre- and post-pregnancy mammary organoids. In doing so, we identified that regions associated with genes controlling Extracellular matrix (ECM) remodeling (3 h), response to lipids (12 h), and wound healing (4 8 h) gained higher levels of H3K27ac mark in post-pregnancy mammary organoids (Fig. 3g). Given that these pathways control tissue remodeling during pregnancy-induced mammary expansion (ECM, 3 h) [47, 48], milk associated components (lipids, 12 h) [49, 50], and involution-like processes (wound healing, 48 h) [51, 52], our analyses further suggests that post-pregnancy organoids treated with pregnancy hormones may have an advanced developmental progress across all stages of pregnancy-induced development.

### Utilization of Organoid Cultures to Define Players in Pregnancy-Induced Development and Post-Pregnancy Epigenome

The identity of factors that establish and maintain the post-pregnancy epigenome remains unclear. In contrast, many factors have been described to play a role during overall pregnancy-induced development of the mammary gland. In fact, loss of the histone methyltransferase factor Enhancer of zest homolog 2 (EZH2), a key component of the Polycomb Repressive Complex 2 (PRC2), has been shown to impact pregnancy-induced stages of mammary gland development and duct elongation defects in vivo [9]. In order to illustrate the robustness of utilizing organoid systems to understand the pregnancy-induced enhancer landscape, we utilized an EZH2 chemical inhibitor (UNC1999) to block its activity in pregnancy hormone treated pre- and post-pregnancy organoids, and characterize its role on branching morphogenesis and *Csn2* mRNA levels.

**Fig. 4:**
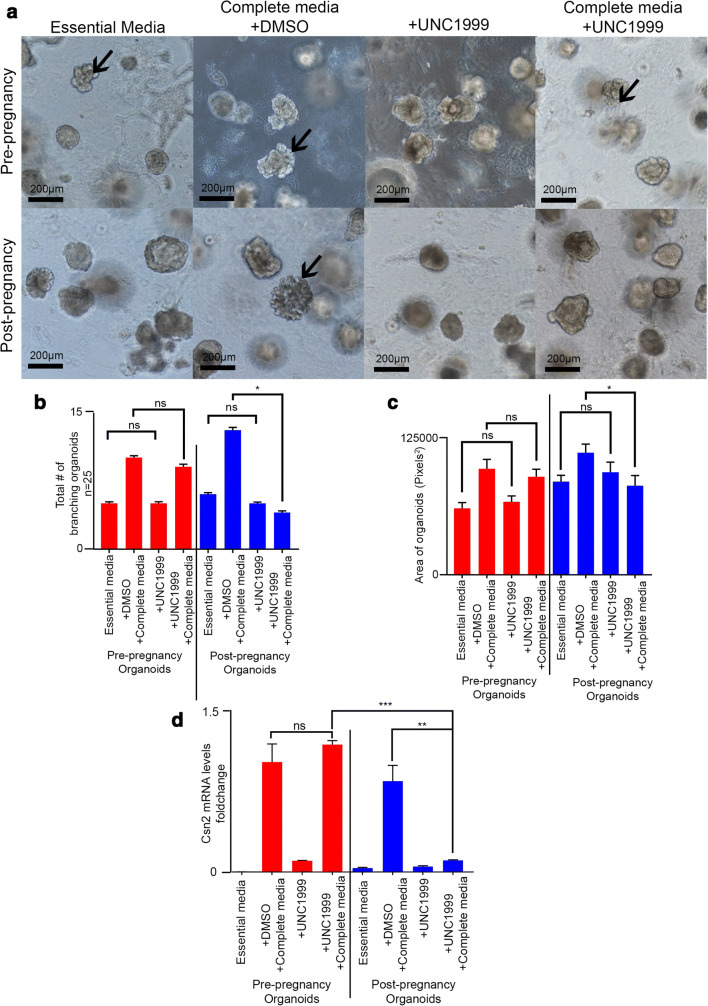
**Utilization of organoid cultures to define players in pregnancy-induced development and post-pregnancy epigenome.** (**a**) Representative brightfield images of mammary organoids treated with essential medium or complete medium, supplemented with either DMSO (Dimethyl sulfoxide, control), or EZH2 inhibitor UNC1999 for 48 h. Arrows indicated examples of branching organoids. Scale = 200 μm. (**b**) Branching quantification of pre- and post-pregnancy mammary organoid cultures treated with essential medium or complete medium, supplemented with either DMSO (control) or EZH2 inhibitor UNC1999. 13 fields of view per well/replicate. *n* = 25 organoids. ns = not significant; *p = 0.027 differences between post-pregnancy organoids treated with complete media and complete media with UNC1999. Error bars indicate standard error of the mean (SEM) across samples of same experimental group. *p* values were defined using Students t-test. (**c**) Size quantification of pre- and post-pregnancy mammary organoid cultures treated with essential medium or complete medium, supplemented with either DMSO (control) or EZH2 inhibitor UNC1999. *n* = 20 organoids per condition. ns = not significant; *p = 0.018 differences between post-pregnancy organoids treated with complete medium and DMSO and post-pregnancy organoids treated with complete medium and UNC1999. For analyses, error bars indicate standard error of the mean (SEM) across samples of same experimental group. *p* values were defined using Welch’s t-test. (**d**) *Csn2* mRNA levels (qPCR) in pre- and post-pregnancy mammary organoid cultures treated with either essential medium or complete medium, with DMSO control or UNC1999. *p = 0.0364 differences between post-pregnancy organoids treated with complete medium and DMSO and post pregnancy organoids treated with complete medium and UNC1999. ***p = 0.0009 differences between pre-pregnancy organoids treated with complete medium and UNC1999, and post-pregnancy organoids treated with complete medium and UNC1999. Error bars indicate standard error of the mean (SEM) across samples of same experimental group. *p* values were defined using Welch’s t-test

We found that EZH2 inhibition had no significant effect on branching morphogenesis of pre-pregnancy mammary organoids, independent of growth conditions (Fig. 4a-b). Conversely, we found that EZH2 inhibition blocked the effects of pregnancy hormones on post-pregnancy mammary organoids with a 3.25-fold decrease in the number of branching organoids, thus suggesting a specific role for EZH2 on controlling the branching of organoids previously exposed to pregnancy hormones (Fig. 4a-b). We also observed EZH2 inhibition had a small, but statistically significant effect on post-pregnancy organoid size, yielding an ~1.35-fold smaller organoids than those grown with complete media treatment without EZH2 inhibition, an effect not observed in pre-pregnancy organoids (Fig. 4c). Moreover, we also observed an ~11-fold downregulation of *Csn2* mRNA in post-pregnancy organoids grown with pregnancy hormones and the EZH2 inhibitor, in comparison to pre-pregnancy organoids under the same conditions (Fig. 4d), further suggesting that EZH2 may work in conjunction with some of the pregnancy-induced epigenetic modifications. Overall, this proof-of-principal analyses illustrates the robustness of organoid systems to understand gene activation and epigenomic reprogramming. In conclusion, we demonstrate that organoid cultures are a suitable system to define specific drivers of gene regulation from a pregnancy-induced landscape perspective.

## Discussion

Our studies demonstrate that organoid cultures are a suitable approach for investigating the effects of pregnancy hormones on the epigenome and transcriptional output of MECs, as well as providing mechanistic insight into how such responses occur in cells previously exposed to a pregnancy cycle in vivo. Utilizing our 3D organoid culturing method to dissect molecular mechanisms controlled by pregnancy signals in MECs presents a series of advantages. For example, the pre-culturing timing of ~6 days, designed to allow mammary organoids to acclimate to culturing, provides a normalization period to remove non-MECs from culture, and to diminish MEC-specific signals present in mice at distinct stages of the estrous cycle. In addition, our proposed method provides a platform to dissect into cell-autonomous mechanisms that operate within a pregnancy-induced epigenome, given that changes to ECM and immune composition have been described in mammary glands after a pregnancy cycle [51, 53–55] . Nonetheless, the incorporation of different ECM substrates and immune cells into organoid cultures may highlight further signaling pathways that together with MECs, influence a myriad of mechanisms controlling pregnancy-induced development.

The present study validated observations proposed in flow cytometry isolated MECs and supports the idea that a full pregnancy cycle induces stable epigenomic changes that alter the transcriptional output of MECs [1]. By analyzing markers of active regulatory regions (H3K27ac), our current method allowed for a snapshot of epigenetic changes in a timely fashion, thus illustrating some of the immediate molecular responses to pregnancy hormones. Given that additional organoid methods can mimic phenotypic alterations observed during involution [6], a deep molecular analysis of such developmental stage would provide further insights into how the epigenome is shaped to assume a post-involution state. Moreover, incorporating additional histone marks of poised enhancers (H3K4me1) or repressed enhancers (H3K27me) will illustrate parts of the epigenome that may became less active after pregnancy. Interestingly, EZH2 has been implicated to contribute to the memory machinery of cells either functioning as or recruiting DNA methyltransferases to certain genes, which could play a part in how post-pregnancy cells turn off certain genes during pregnancy [56]. Specifically, mammary epithelial cells from pre-pregnancy mice lacking EZH2 express high levels of milk related genes Csn2 and Whey acidic protein (Wap) earlier in pregnancy than wild-type mice [57], supporting its specialized role on controlling an epigenomic landscape brought by a previous pregnancy cycle. Further investigation regarding the relationship between EZH2, epigenetic memory, and gene re-activation, in response to pregnancy hormones, is needed to precily  define molecular dynamics that control mammary development and parity-induced epigenome.

The application of an organoid strategy could also help to define the effects that the age of pregnancy has on controlling molecularly relevant and evolutionary conserved epigenetic modifications, which govern post-pregnancy breast tissue homeostasis and development in consecutive pregnancies. For example, the age of first pregnancy in women has a strong influence on milk supply and breast cancer development. Even though several studies report that human females have a significantly increased milk supply during a second pregnancy [58–61], women that experienced their first pregnancy after 35 years of age are at risk to require medical intervention to improve milk production and breastfeeding [62]. Thus, employing tissue fragments collected from women and rodents spanning a variety of age of first pregnancy and those from aged subjects never exposed to pregnancy hormones will address outstanding questions about the impact of aging on evolution and molecular adaptation of breast cells to control development, gene expression and milk production in response to pregnancy signals.

In addition, a series of large-scale population studies found correlations between the age of first full-term pregnancy and breast cancer development [63, 64]. Women younger than the age of 25 have an approximate 30% decrease in the incidence of breast cancer. In contrast, pregnancy in women older than 38 years of age correlates with a 30–50% increase in developing more aggressive subtypes of breast cancer within the first ten years after giving birth [63, 65]. Notably, we recently reported that a full pregnancy cycle blocked cancer initiation and epigenetic reprograming in murine MECs after overexpressing the potent oncogene cMYC, suggesting that pregnancy-induced molecular changes may impact the transcriptional output that can drive cancer initiation [1]. Such effects were also observed in organoid cultures derived from post-pregnancy MECs, which supports the conclusion that cell autonomous signals that block cancer initiation can also be studied in 3D culturing systems.

Lastly, we provided a proof-of-principal perspective regarding the robustness of organoid systems. The incorporation of small chemical inhibitors, or more direct genetic manipulation of regulatory factors, to the culturing system presented here may also represent a cost- and time-effective screening strategy to define new players that control the mammary epithelial epigenome and block cancer initiation. Such strategies could also be employed in organoid cultures derived from healthy breast tissue of women with genetic or familial predisposition to develop breast cancer, as an additional tool to search for strategies that may prevent on further decrease the risk of cancer.

## Methods

**Antibodies.** All antibodies were purchased from companies listed below and used without further purification. Antibodies for IF: anti-β-casein/csn2 mouse monoclonal antibody (Santa Cruz Biotechnology Inc., #sc-166,530, 200 μg/mL, 1:300 dilution, RRID: AB_2084348), anti-Cytokeratin5 rabbit monoclonal antibody (Abcam, #EP1601Y, 0.5 mg/ML 1:300 dilution RRID:AB_869890), anti-Cytokeratin-8 mouse monoclonal antibody (Abcam, #EP1628Y, 0.5 mg/mL 1:300 dilution, RRID:AB_869901). Antibody for Cut&Run: H3K27ac histone marks (Abcam, #ab4729, RRID:AB_2118291).

### Isolation of Primary Mammary Epithelial Organoids

Primary mammary organoids were derived from either pre-pregnant or post-pregnant female Balb/c mice as previously described [2]. In short, pipettes and tubes were pre-coated with 5% BSA solution (in 1X PBS, Gibco #A10008–01). Female mice (~15 weeks old) were euthanized via CO_2_ asphyxiation, and mammary glands from 3 animals (thoracic and inguinal mammary glands pairs) were removed and collected into 10 cm culture dishes. The pooled glands were then minced under sterile conditions using scalpels, cutting around 50 times in a crisscross pattern to loosen the tissue of the glands. The minced glands were then transferred to a 50 mL falcon tube that contained 20 mL of collagenase solution which consisted of AdDf+++ (Advanced DMEM F12 (Dulbecco’s Modified Eagle Medium/F-1,5 mM GlutaMax 5 mM HEPES, 1x Penicillin/Streptomycin), FBS (1%, Corning #35–010-CV), Insulin (5μg/mL, Sigma #I9278) and Collagenase A (2 mg/mL, type IV from Clostridium histolyticum, Sigma #C5138). The tubes with the glands and collagenase solution were shaken at 200 RPM for 30–40 min until solution became cloudy, and large chunks of tissue dissipated. After digestion, 1 mL of FBS was added, and then solution was passed up and down a 5 mL pipette 10 times to ensure complete disassociation of the tissue. The solution was spun down in a centrifuge at 300 x g for 5 min at room temperature and the fat-containing supernatant was removed. The pellet was resuspended in 10 mL of AdDf+++ and then passed through a 100 μm strainer (Falcon #352360) to ensure no large tissue pieces would proceed. An additional room temperature 5-min centrifugation step at 300 x g was used to wash any remaining enzyme from the pelleted epithelium. The pellet was then resuspended in 10 mL of AdDf+++, pulse centrifugated to 500 x g, then had supernatant removed. This was repeated a total of 3 times.

### Organoid Cultures

The organoid pellet was resuspended using a pre-chilled pipette tip in desired amount of Matrigel (100%, Corning #354230). In a 37 °C pre-warmed 24 well plate, three small domes were made using a total of 50 μl of Matrigel in a triangle-shaped pattern. The plates were then inverted and placed into a CO_2_ incubator (5% CO_2_, 37 °C) for 20 min to allow Matrigel to solidify. Each of the wells was filled with 0.5 mL of Essential organoid medium AdDf+++, supplemented with 1x ITS (Insulin/Transferrin/Sodium Selenite, Gibco #41400–045) and FGF-2 (Final concentration: 5 nm, PeproTech #450–33) for 6 days. Medium was changed every two days. The cultured mammary organoids were then grown in medium lacking FGF2 for 24 h and then incubated with complete medium (AdDf+++, supplemented with ITS (Final Concentration:1x, Insulin/Transferrin/Sodium Selenite, Gibco, #41400–045), 17-β-Estradiol (Final concentration: 40 ng/mL, Sigma #E2758), Progesterone (Final concentration: 120 ng/mL, Sigma #P8783), Prolactin (Final concentration: 120 ng/mL,Sigma #L4021). Organoids were isolated from Matrigel using Cell Recovery solution (0.5 mL, Corning #354253).

### Organoid Branching and Size Quantification

Organoid visualization and image collection were performed on a Nikon Eclipse TI microscope utilizing NIS-Elements BR software (Nikon). For branching and size quantification, at least 13 fields of view and 20 to 25 organoids were analyzed. The area (size) of mammary organoids in each image was measured via ImageJ. A branching classification was given to organoids displaying three or more elongated buds.

### RNA Isolation and RT-qPCR

Medium was removed from each of the wells and then washed with 0.5 mL 1x PBS. RNA was extracted by adding Trizol (0.5 mL, Thermo Fisher Scientific, #15596018) to each well with organoids. Reverse transcription was carried out using SuperScript III ™ kit (Thermo Fisher Scientific). RTqPCR was performed using a Quantstudio 6 with SYBR Green Master mix (Applied Biosystems #4368577). Each reaction was run in at least duplicate. Relative gene expression was calculated via the deltadeltact method in which the values for the measured genes were normalized to the house keeping gene, mouse *β-actin* mRNA. Primers sequences targeting mRNA of *β-actin*, *Csn2* and *Csn3* genes were designed as previously described [1].

### Mammary Organoids Wholemount Immunofluorescence Staining

Each well was washed with 0.5 mL of 1X PBS, followed by the addition of Cell recovery solution (0.5 mL, Corning #354253). Culture plates were them incubated at 4 °C for 30 min or until Matrigel domes were no longer visible, followed by the addition of 1 mL of AdDf+++ and gentle pipetting all organoids were released from Matrigel. Organoids were then transferred to a 1% BSA (in 1X PBS, Gibco #A10008–01) pre-coated 15 mL conical tube, and spun at 500 x g for 5 min at room temperature (RT). Organoid pellets were gently resuspended in 1 mL of 4% PFA (in 1X PBS, Electron Microscopy Sciences, #15711) for 1 h at RT, following centrifugation at 500 x g for 5 min at RT. Fixed organoids were permeabilized with 1 mL of Permeabilization solution (0.5% Triton X-100, Sigma #93443 in 1X PBS) and incubated for 30 min at RT, followed by centrifugation at 500 x g for 5 min at RT. Permeabilized organoids were washed once with 1 mL of washing solution (0.1% Tween-20 solution, MP, #9005-64-5 in 1X PBS), followed by centrifugation at 500 x g for 5 min at RT. Fixed and permeabilized organoid pelleted were gently resuspended with 0.5 mL of blocking buffer (1X PBS; 300 mM Glycine, Fischer-Scientific, #G45–212; 10 mg/mL BSA, Sigma #A2153; 5% Goat serum, Abcam #ab7481) and incubated for 1 h at RT, followed by centrifugation at 500 x g for 5 min at RT. Blocked organoids were then resuspended in blocking buffer containing indicated concentrations of antibodies, and incubated overnight at 4 °C with constant agitation. Staining solution was removed, and organoids were washed with 1 mL of washing solution, centrifugation at 500 x g for 5 min at RT for a total of 3 times. Stained organoids were then stained with Propidium Iodide (PI, for nuclear staining, Invitrogen, 1:1000 dilution), at room temperature for 15 min. Staining solution was removed, and organoids were washed with 1 mL of washing solution and then underwent centrifugation at 500 x g for 5 min at RT for a total of 3 times. At the end of the last centrifugation, the supernatant was removed, and one drop of Prolong™ Glass Antifade Mountant (Invitrogen #P36982) was added to stained organoids, followed by mounting on a glass slide and allowed to cure overnight in the dark. All imaging was acquired on a Zeiss 780 Confocal Microscope.

### RNA-Seq Library Preparation and Analyses

Mammary organoids (*n* = 2–3 wells per technical replicate) were collected, dissociated and resuspended in Trizol (Thermo Fisher Scientific, #10296010) for RNA extraction. Double stranded cDNA synthesis and Illumina libraries were prepared utilizing the Ovation RNA-seq system (V2) (Nugen Technologies, #7102–32). RNA-seq libraries were prepared utilizing the Ovation ultralow DR multiplex system (Nugen Technologies, #0331–32). Each library (*n* = 2 per experimental condition) was barcoded with Illumina True-seq adaptors to allow sample multiplexing, followed by sequencing on an Illumina NextSeq500, 76 bp single-end run. Bioinformatics analyses were performed with command-line interfaced tools such as FastQC [66] for quality control and Trimmomatic [67] for sequence trimming. We used STAR [68] for mapping reads and deepTools [69] for principal component analysis. Further, we utilized FeatureCounts [70] for assigning reads to genomic features and DESeq [71] to assess changes in expression levels. Gene Set Enrichment Analysis (GSEA) was used for global analyses of differentially expressed genes [72]. For the parity signature analysis (total of 47 genes [18]), differentially expressed genes above 0.5 log2foldchange were counted as upregulated, while genes below −0.5 log2foldchange were counted as downregulated.

### Cut&Run Library Preparation and Analyses

Mammary organoids (n = 2–3 wells per technical replicate) were collected, dissociated, and permeabilized with digitonin, following overnight incubation with antibody against H3K27ac histone marks (Abcam, #ab4729) at 4°C with constant agitation. Antibody-chromatin complexes were fragmented with pA-MNAse and purified utilizing Phenol-Chloroform. Cut&Run libraries (n = 2 per experimental condition) were amplified and barcoded using Clontech DNA Smart ChIP-Seq kit (Clontech, #634866) in accordance with the manufacturer’s instructions, then pooled for sequencing on an Illumina NextSeq500, 76 bp paired-end run. Reads were mapped to the indexed mm9 genome using bowtie2 short-read aligner tool [73] using default settings. Sparse Enrichment Analysis for Cut&Run (SEACR) peak-calling program [74] was used to identify enriched genomic regions with an empirical threshold of *n* = 0.01, returning the top n fraction of peaks based on total signal within peaks. The stringent argument was implemented, which used the summit of each curve. Further downstream analyses were performed using various command-line interfaced programs including deepTools for investigating H3K27ac peak intensity at DNA binding motifs and bedtools [75] for defining regions that are shared between conditions as well as peaks exclusive to each condition. Open source software such as Enrichr [76, 77] for comparing peaks against publicly available data and GREAT [78] for gene ontology analyses served to support condition-exclusive analyses. Additionally, UCSC’s Genome Browser [79] was used to investigate region specific H3K27ac peak intensity. H3K27ac Cut&Run peaks were utilized as input for an unbiased transcription factor analyses using Analysis of Motif Enrichment (AME) [80] and Find Individual Motif Occurrences (FIMO) [81] was used to computationally define DNA binding motif regions. DESeq2 [82] was utilized to generate genomic regions with differential H3K27ac levels sample groups (FDR < 0.05).

## Supplementary Information

ESM 1 (DOCX 2185 kb)

## Abbreviations

3D: Three-dimensional
AdDf+++: Advanced DMEM F12 (Dulbecco’s modified eagle medium/F-1,5 mM glutaMax 5 mM HEPES, 1× penicillin/streptomycin)
AME: Analysis of motif enrichment
β-actin: Beta actin
BSA: Bovine serum albumin
Csn1s2a: Casein alpha s2-like A
cDNA: Complementary DNA
ChIP-seq: Chromatin immune precipitation sequencing
Cited1: Cbp/P300 interacting transactivator with Glu/Asp Rich Carboxy-Terminal Domain 1
cMYC: Cellular myelocytomatosis
CO_2_: Carbon dioxide
Csn2: Casein 2
Csn3: Casein 3
Cut&Run: Cleavage under targets and release using nuclease
DEGs: Differentially expressed genes
DESeq: Program for differential gene expression analysis
DMSO: Dimethyl sulfoxide
eBOX: cMYC motif
ECM: Extracellular matrix
Elf5: E74-like factor 5 gene
ERα: Estrogen Receptor alpha
EREs: ER alpha motif
EZH2: Enhancer of zest homolog 2
FastQC: Program for quality control check of high throughput sequencing dataset
FBS: Fetal bovine serum
FDR: False discovery rate
FGF2: Fibroblast growth factor 2
FIMO: Find individual motif occurrences
FOXC2: Forkhead box protein C2
GAS: STAT5 motif
GO: Gene ontology
GSEA: Gene set enrichment analysis
GREAT: Program for annotation of sequencing dataset
H3K27ac: Histone 3 lysine 27 acetylation
H3K4me1: Histone 3 lysine 4 monomethylation
H4K27me3: Histone 4 lysine 27 trimethylation
Hrs: hours
IF: Immunofluorescence
IRF1: Interferon responsive factor 1
ITS: Insulin/Transferrin/Sodium Selenite
KRT5: Cytokeratin 5
KRT8: Cytokeratin 8
MECs: Mammary epithelial cells
mRNA: messenger RNA
NCBI: National Center for Biotechnology Information
ns: not significant
pA-MNAse: fusion protein produced by the pK19-pA-MN plasmid
PBS: Phosphate buffered saline
PC-1: Principal Component 1
PC-2: Principal Component 2
PFA: Paraformaldehyde
PI: Propidium Iodide
RNA-seq: RNA sequencing
PRC2: Polycomb repressive complex 2
Prlr: Prolactin receptor
RPM: revolutions per minute
RT: Room temperature
RT-qPCR: Real time quantitative polymerase chain reaction
SEACR: Sparse enrichment analysis for cut & run
SEM: Standard error of the mean
SP3: Specificity protein 3
STAR: Program for mapping sequencing dataset
STAT5: Signal transducer and activator of transcription 5
TF: Transcription factor
UCSC: University of California, Santa Cruz
UNC1999: Chemical inhibitor of EZH2
Vdr: Vitamin D receptor
Wap: Whey acidic protein
× g: times gravity
