# Supplementary material for: Characterization of Organoid Cultures to Study the Effects of Pregnancy Hormones on the Epigenome and Transcriptional Output of Mammary Epithelial Cells
Source: J Mammary Gland Biol Neoplasia. 2020 Nov 1;25(4):351–66. doi: 10.1007/s10911-020-09465-0 (PMC7960614; doi:10.1007/s10911-020-09465-0)
Supplement: Supplementary file 1 — (DOCX 2185 kb) [file 10911_2020_9465_MOESM1_ESM.docx]

**Supplementary Figure S1. Organoid culture scheme.** Experimental design flowchart of derivation of mammary organoids, Matrigel dome layout, culture treatment, and downstream organoid applications.

**
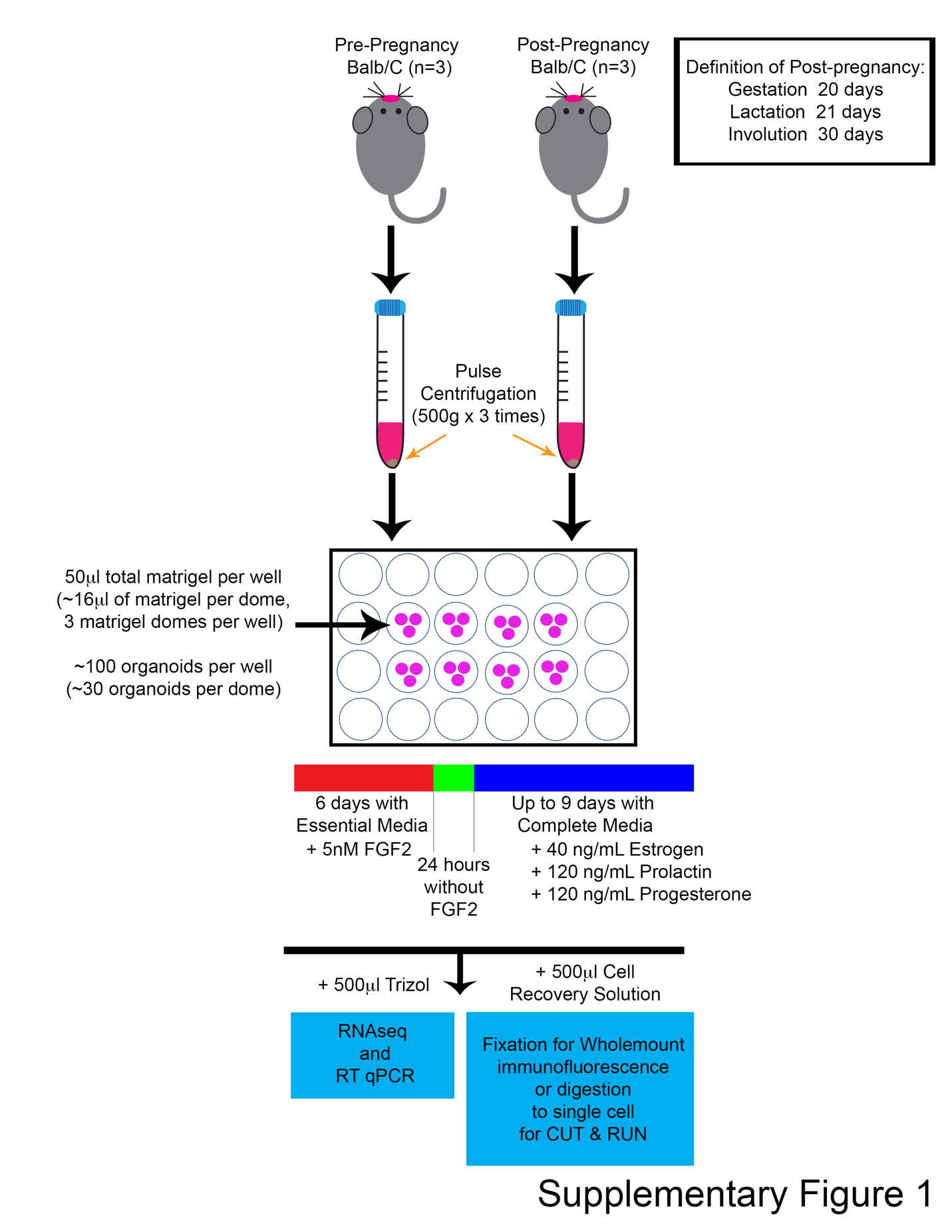
**

**Supplementary Figure S2. Pregnancy hormones induce changes to the epigenome of pre-pregnancy mammary organoids.**  (**A**) Classification of total H3K27ac peaks from pre-pregnancy organoids before (top left) and after complete medium treatment for 3 hrs (top right), 12 hrs (bottom left) and 48 hrs (bottom right) according to their genomic distribution. (**B**) Genome browser tracks showing distribution of H3K27ac peaks in pre-pregnancy organoids before and after complete medium treatment for 3 hrs for the Cited1 locus, pre-pregnancy organoids before and after complete medium treatment for 12 hrs for the Vdr locus and pre-pregnancy organoids before and after complete medium treatment for 48 hrs for the Csn1s2a locus. (**C**) Gene ontology (GO) term analyses enriched in H3K27ac peaks exclusive to pre-pregnancy organoids at 3 hrs (top panel), 12hrs (middle panel) and 48hrs (bottom panel) complete medium treatment, and Gene ontology analyses for overlapping peaks of 3 hrs, 12 hrs and 48 hrs (fourth from top). (**D**) Venn diagrams comparing exclusive H3K27ac peaks pre-pregnant mammary organoid cultures after 3 hrs,12 hrs and 48 hrs treatment with complete medium. (**E**) Bar plot of unbiased DNA motif analyses in H3K27ac peaks exclusive to pre-pregnancy organoids after 3 hrs, 12 hrs and 48 hrs treatment with complete medium.

**
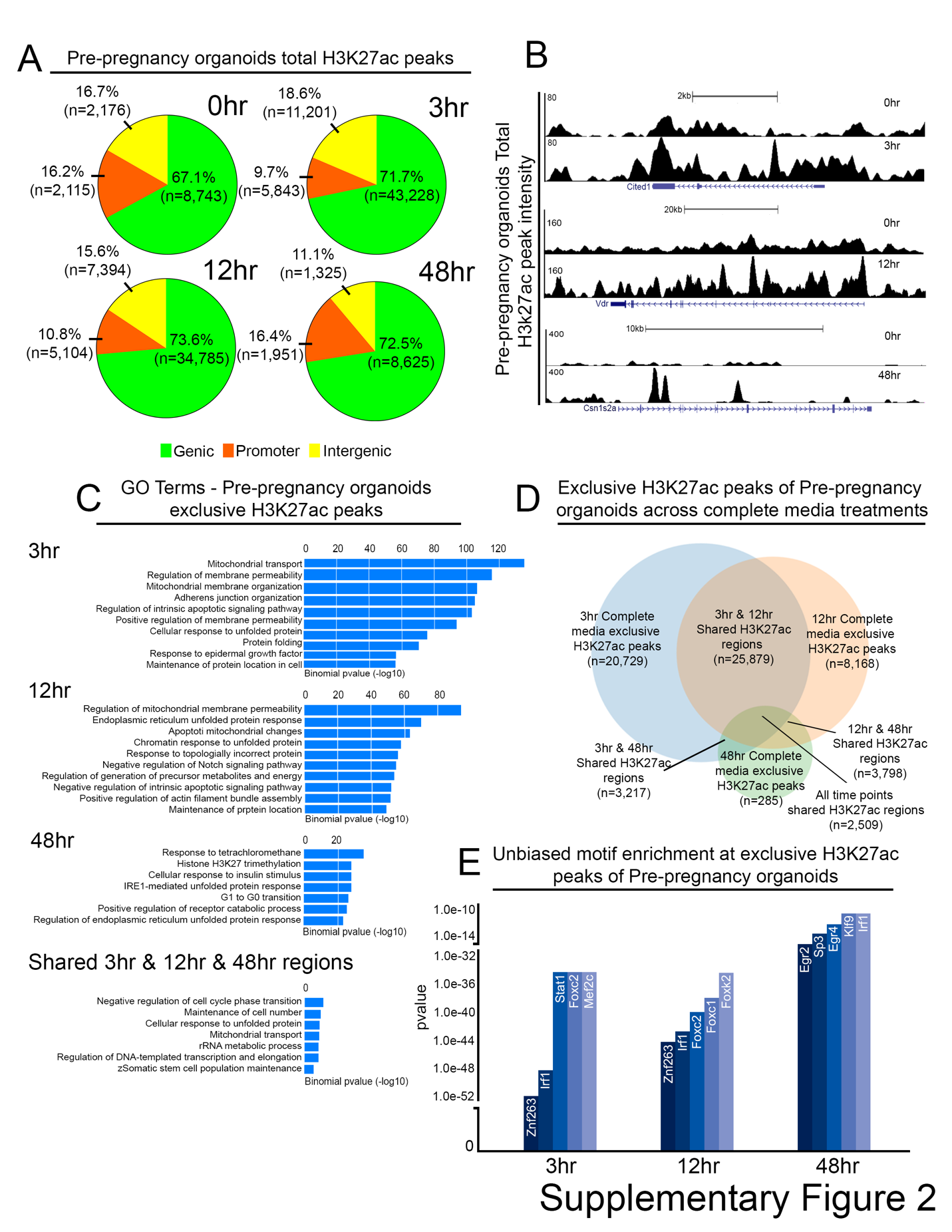
**

**
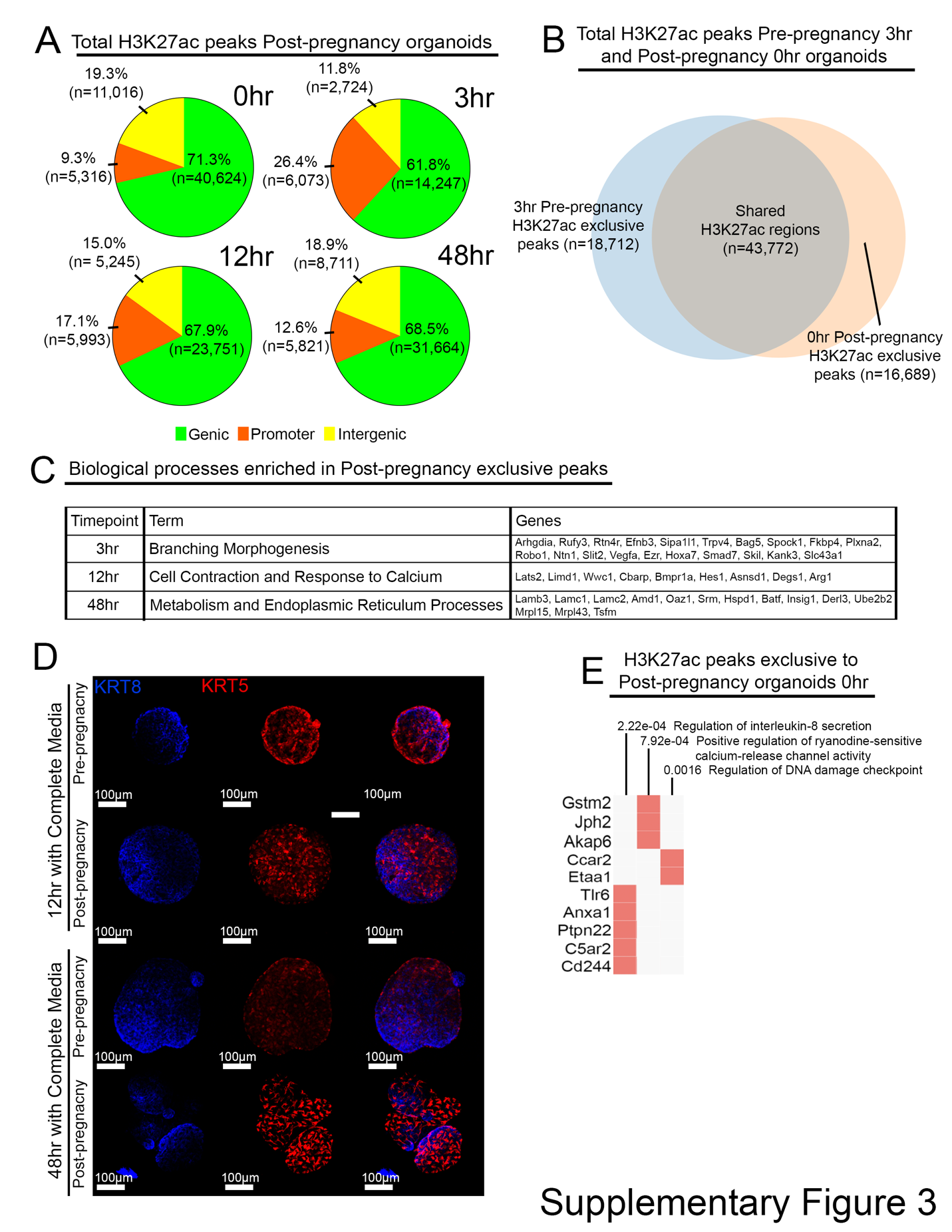
**

**Supplementary Figure S3. Pregnancy hormones induce specific changes to the epigenome of post-pregnancy mammary organoids.** (**A**) Classification of total H3K27ac peaks from post-pregnancy organoids before (top left) and after complete medium treatment for 3 hrs (top right), 12hrs (bottom left) and 48hrs (bottom right) according to their genomic distribution. (**B**) Venn diagram comparing exclusive H3K27ac peaks of pre-pregnancy organoids after 3hrs treatment with complete medium and untreated post-pregnancy organoids. (**C**) Table displaying biological processes enriched in post-pregnancy organoids treated with complete at 3 hrs, 12 hrs and 48 hrs with complete medium. (**D**) Immunofluorescence (IF) imaging of whole mounted pre-and post-pregnancy Balb/c organoids after complete medium for both 12 hrs and 48 hrs, visualizing, KRT8 (blue), KRT5 (red). Scale: 100μm. (**E**) Heatmap displaying processes and genes enriched in untreated post-pregnancy organoids.

**Supplemental Tables**

**Supplementary Table S1.** List of genes identified as part of Parity induced signature, with differential expression analysis (Log2foldchange) comparing mRNA levels between pre-pregnancy organoids grown with essential medium (-log2foldchange) or complete medium (log2foldchange). In bold = genes that agree with differential expression proposed by the original description of the signature.

| Gene ID | Signature according to Blakely et al [18] | mRNA log2FoldChange - mammary organoids |
| --- | --- | --- |
| CCL5 | Up regulated in post-pregnancy mammary tissue | -2.475294415 |
| **IDB1** | **Down regulated in post-pregnancy mammary tissue** | **-1.768053318** |
| **CS3** | **Down regulated in post-pregnancy mammary tissue** | **-1.752754864** |
| **IDB3** | **Down regulated in post-pregnancy mammary tissue** | **-1.051084248** |
| IGF1 | Up regulated in post-pregnancy mammary tissue | -1.024458256 |
| IGFBP5 | Up regulated in post-pregnancy mammary tissue | -0.999107314 |
| **AKAP12** | **Down regulated in post-pregnancy mammary tissue** | **-0.86562153** |
| **LGALS1** | **Down regulated in post-pregnancy mammary tissue** | **-0.844458286** |
| TGFB3 | Up regulated in post-pregnancy mammary tissue | -0.822762793 |
| MMP12 | Up regulated in post-pregnancy mammary tissue | -0.67023901 |
| **SPARC** | **Down regulated in post-pregnancy mammary tissue** | **-0.614758705** |
| SLC3A2 | Up regulated in post-pregnancy mammary tissue | -0.592413036 |
| **ENPP2** | **Down regulated in post-pregnancy mammary tissue** | **-0.571810124** |
| **FSTL1** | **Down regulated in post-pregnancy mammary tissue** | **-0.553754504** |
| **COL5A2** | **Down regulated in post-pregnancy mammary tissue** | **-0.546700179** |
| **GHR** | **Down regulated in post-pregnancy mammary tissue** | **-0.533360882** |
| FN1 | Down regulated in post-pregnancy mammary tissue | -0.484101883 |
| LCN2 | Up regulated in post-pregnancy mammary tissue | -0.381344929 |
| LUM | Down regulated in post-pregnancy mammary tissue | -0.373070993 |
| MOD1 | Down regulated in post-pregnancy mammary tissue | -0.346744166 |
| TMSB10 | Down regulated in post-pregnancy mammary tissue | -0.322705468 |
| TUBA1 | Down regulated in post-pregnancy mammary tissue | -0.314822781 |
| ALDOC | Up regulated in post-pregnancy mammary tissue | -0.289929762 |
| CAT | Up regulated in post-pregnancy mammary tissue | -0.227224589 |
| TPM1 | Down regulated in post-pregnancy mammary tissue | -0.100298654 |
| PDE4B | Up regulated in post-pregnancy mammary tissue | 0.051 |
| ANXA5 | Down regulated in post-pregnancy mammary tissue | 0.02391884 |
| CYR61 | Down regulated in post-pregnancy mammary tissue | 0.071325815 |
| CAR2 | Up regulated in post-pregnancy mammary tissue | 0.150496424 |
| PSMC2 | Down regulated in post-pregnancy mammary tissue | 0.181277377 |
| HDAC2 | Down regulated in post-pregnancy mammary tissue | 0.227159109 |
| CD74 | Down regulated in post-pregnancy mammary tissue | 0.253126378 |
| CYP4B1 | Down regulated in post-pregnancy mammary tissue | 0.253255619 |
| TUBB5 | Up regulated in post-pregnancy mammary tissue | 0.254169528 |
| SLC25A4 | Down regulated in post-pregnancy mammary tissue | 0.33186431 |
| SPRX | Down regulated in post-pregnancy mammary tissue | 0.43723039 |
| SLC11A2 | Down regulated in post-pregnancy mammary tissue | 0.540467033 |
| **PIGR** | **Up regulated in post-pregnancy mammary tissue** | **0.60881789** |
| PGK1 | Down regulated in post-pregnancy mammary tissue | 0.718172102 |
| **MCPT2** | **Up regulated in post-pregnancy mammary tissue** | **1** |
| CES3 | Down regulated in post-pregnancy mammary tissue | 1 |
| AREG | Down regulated in post-pregnancy mammary tissue | 1.127749544 |
| CDO1 | Down regulated in post-pregnancy mammary tissue | 2.599215109 |
| CITED1 | Down regulated in post-pregnancy mammary tissue | 3.654852431 |
| **GLYCAM1** | **Up regulated in post-pregnancy mammary tissue** | **6.63599128** |
| **CSNG** | **Up regulated in post-pregnancy mammary tissue** | **8.928605885** |
| **CSNB** | **Up regulated in post-pregnancy mammary tissue** | **11.23867885** |

**Supplementary Table S2.** List of genes identified as part of Parity induced signature, with differential expression analysis (Log2foldchange) comparing mRNA levels between pre-pregnancy organoids (-log2foldchange) and post-pregnancy organoids (log2foldchange), grown with essential medium. In bold = genes that agree with differential expression proposed by the original description of the signature.

| Gene ID | Signature according to Blakely et al [18] | mRNA log2FoldChange - mammary organoids |
| --- | --- | --- |
| **CS3** | **Down regulated in post-pregnancy mammary tissue** | **-4.752652529** |
| **CD74** | **Down regulated in post-pregnancy mammary tissue** | **-1.618818856** |
| **ENPP2** | **Down regulated in post-pregnancy mammary tissue** | **-1.33994045** |
| **LGALS1** | **Down regulated in post-pregnancy mammary tissue** | **-1.319340976** |
| **TUBA1** | **Down regulated in post-pregnancy mammary tissue** | **-1.31531605** |
| CCL5 | Up regulated in post-pregnancy mammary tissue | -1.223995339 |
| **IDB1** | **Down regulated in post-pregnancy mammary tissue** | **-1.0847429** |
| **AREG** | **Down regulated in post-pregnancy mammary tissue** | **-0.884841109** |
| **FSTL1** | **Down regulated in post-pregnancy mammary tissue** | **-0.68911381** |
| IGF1 | Up regulated in post-pregnancy mammary tissue | -0.679706716 |
| **CYR61** | **Down regulated in post-pregnancy mammary tissue** | **-0.62793218** |
| **SPARC** | **Down regulated in post-pregnancy mammary tissue** | **-0.525586619** |
| **CITED1** | **Down regulated in post-pregnancy mammary tissue** | **-0.517144566** |
| **FN1** | **Down regulated in post-pregnancy mammary tissue** | **-0.455163482** |
| GLYCAM1 | Up regulated in post-pregnancy mammary tissue | -0.451480035 |
| LCN2 | Up regulated in post-pregnancy mammary tissue | -0.435308223 |
| COL5A2 | Down regulated in post-pregnancy mammary tissue | -0.381490883 |
| CDO1 | Down regulated in post-pregnancy mammary tissue | -0.367795667 |
| SLC11A2 | Down regulated in post-pregnancy mammary tissue | -0.364332426 |
| TMSB10 | Down regulated in post-pregnancy mammary tissue | -0.352825789 |
| ANXA5 | Down regulated in post-pregnancy mammary tissue | -0.295782571 |
| TUBB5 | Up regulated in post-pregnancy mammary tissue | -0.291862419 |
| PDE4B | Up regulated in post-pregnancy mammary tissue | -0.281075231 |
| TPM1 | Down regulated in post-pregnancy mammary tissue | -0.197406329 |
| SPRX | Down regulated in post-pregnancy mammary tissue | -0.086782535 |
| TGFB3 | Up regulated in post-pregnancy mammary tissue | -0.073164495 |
| PSMC2 | Down regulated in post-pregnancy mammary tissue | -0.066809849 |
| IDB3 | Down regulated in post-pregnancy mammary tissue | -0.053135263 |
| IGFBP5 | Up regulated in post-pregnancy mammary tissue | -0.038838079 |
| MMP12 | Up regulated in post-pregnancy mammary tissue | -0.025502923 |
| GHR | Down regulated in post-pregnancy mammary tissue | 0.007682631 |
| SLC3A2 | Up regulated in post-pregnancy mammary tissue | 0.018680039 |
| AKAP12 | Down regulated in post-pregnancy mammary tissue | 0.045970127 |
| HDAC2 | Down regulated in post-pregnancy mammary tissue | 0.108813843 |
| SLC25A4 | Down regulated in post-pregnancy mammary tissue | 0.165440487 |
| ALDOC | Up regulated in post-pregnancy mammary tissue | 0.222925903 |
| LUM | Down regulated in post-pregnancy mammary tissue | 0.315969375 |
| **CAT** | **Up regulated in post-pregnancy mammary tissue** | **0.377485947** |
| MOD1 | Down regulated in post-pregnancy mammary tissue | 0.505766174 |
| **CAR2** | **Up regulated in post-pregnancy mammary tissue** | **0.659755422** |
| CYP4B1 | Down regulated in post-pregnancy mammary tissue | 0.776213815 |
| **PIGR** | **Up regulated in post-pregnancy mammary tissue** | **0.920427058** |
| **MCPT2** | **Up regulated in post-pregnancy mammary tissue** | **1** |
| CES3 | Down regulated in post-pregnancy mammary tissue | 1 |
| PGK1 | Down regulated in post-pregnancy mammary tissue | 1.09677443 |
| **CSNG** | **Up regulated in post-pregnancy mammary tissue** | **3.868408555** |
| **CSNB** | **Up regulated in post-pregnancy mammary tissue** | **6.59312998** |

**Supplementary Table S3.** List of Differentially Expressed Genes (DEGs from analyses comparing mRNA levels between pre-pregnancy organoids (-log2foldchange) and post-pregnancy organoids (log2foldchange) grown with pregnancy hormones, that associate with H3K27ac peaks.

| H3K27ac peak associated genes | mRNA log2FoldChange |
| --- | --- |
| EFNA3 | -4.464276412 |
| UBALD2 | -4.437120349 |
| STK24 | -4.201509566 |
| NLRX1 | -4.105426138 |
| EIF3B | -4.028557057 |
| GRAMD1A | -3.919763596 |
| MPP6 | -3.839189648 |
| DMP1 | -3.838257606 |
| CCDC122 | -3.755149927 |
| 2410022M11RIK | -3.707575375 |
| AICDA | -3.707071603 |
| IRX6 | -3.630706295 |
| LRRC42 | -3.569305097 |
| RBM12B2 | -3.569305097 |
| TPM1 | -3.569305097 |
| NR2F6 | -3.569305097 |
| P3H2 | -3.569305097 |
| AW549542 | -3.569305097 |
| MDK | -3.569305097 |
| SCARNA17 | -3.521912834 |
| RTN4IP1 | -3.51713804 |
| GPSM3 | -3.460132864 |
| CAR6 | -3.325281874 |
| SMIM23 | -3.292537876 |
| RAP2A | -3.291591463 |
| ATF2 | -3.248846724 |
| BPIFB4 | -3.201509566 |
| MIR1966 | -3.201509566 |
| GM9199 | -3.171454334 |
| EDC3 | -3.171454334 |
| CD180 | -3.144220009 |
| NRXN2 | -3.105426138 |
| COX19 | -3.105426138 |
| STK35 | -3.105426138 |
| 1110059G10RIK | -3.105426138 |
| ZFP433 | -3.105426138 |
| TAF2 | -3.105426138 |
| CD59B | -3.105426138 |
| PLCB2 | -3.105426138 |
| PPIL3 | -3.105426138 |
| GAS2L3 | -3.105426138 |
| PPP2CA | -3.105426138 |
| PAIP2B | -3.105426138 |
| CALR4 | -3.105426138 |
| RSPH9 | -3.105426138 |
| FOXJ2 | -3.105426138 |
| GC | -3.105426138 |
| MIR874 | -3.105426138 |
| NXPE4 | -3.105426138 |
| TALDO1 | -3.105426138 |
| GP1BA | -3.105426138 |
| SPACA4 | -3.078994949 |
| CHL1 | -2.988913052 |
| DYNC1H1 | -2.971297435 |
| SHROOM1 | -2.958079335 |
| CHMP3 | -2.958079335 |
| ZDHHC19 | -2.958079335 |
| SRARP | -2.951244046 |
| TMEM53 | -2.8937407 |
| MIR101A | -2.892829624 |
| TBPL1 | -2.892829624 |
| EPGN | -2.856220477 |
| GADD45A | -2.854906987 |
| MYCL | -2.846072474 |
| UCP3 | -2.815960543 |
| HACD3 | -2.772336663 |
| GM525 | -2.745575406 |
| TET2 | -2.707575375 |
| ZBTB5 | -2.707575375 |
| CARD9 | -2.707575375 |
| MPHOSPH8 | -2.707575375 |
| EXTL3 | -2.707575375 |
| MTHFSD | -2.707575375 |
| SLC20A2 | -2.707575375 |
| TEN1 | -2.707575375 |
| PADI3 | -2.707575375 |
| LETMD1 | -2.707315082 |
| MRPS16 | -2.707167082 |
| EIF4E3 | -2.707167082 |
| SEC23IP | -2.706917836 |
| 0610012G03RIK | -2.706628963 |
| SFTPB | -2.706628963 |
| HARBI1 | -2.706628963 |
| WASHC2 | -2.706628963 |
| LRG1 | -2.706628963 |
| TBC1D2 | -2.706628963 |
| GM6297 | -2.706628963 |
| CREB3L1 | -2.706628963 |
| CDK19 | -2.706628963 |
| KIF13A | -2.706628963 |
| EIF4ENIF1 | -2.706628963 |
| MORN3 | -2.706628963 |
| PAPOLG | -2.639600321 |
| OLFR324 | -2.625814649 |
| SEC24D | -2.625814649 |
| ANKRD12 | -2.616677962 |
| PPP2R2A | -2.616547065 |
| PTPRE | -2.569305097 |
| LARP1 | -2.569305097 |
| SHC1 | -2.569305097 |
| 4930451G09RIK | -2.569305097 |
| ERI3 | -2.569305097 |
| GM16938 | -2.542553413 |
| MIR6990 | -2.527276221 |
| PPP5C | -2.522432756 |
| 9130019O22RIK | -2.521912834 |
| MAP7D1 | -2.520463637 |
| KIF9 | -2.513293872 |
| SAP130 | -2.494032449 |
| CD68 | -2.494032449 |
| TMEM130 | -2.464276412 |
| ZFP9 | -2.459094333 |
| CCDC191 | -2.456808307 |
| PPP1R15B | -2.446954802 |
| PLCD1 | -2.44617176 |
| PROM2 | -2.427003807 |
| NPM3 | -2.425325633 |
| KLHL26 | -2.423285444 |
| DGKD | -2.413850639 |
| SLC25A19 | -2.393729583 |
| LCN2 | -2.375310689 |
| RPGR | -2.356708583 |
| PAM16 | -2.356708583 |
| GM20517 | -2.356708583 |
| ANKRD39 | -2.356708583 |
| AFF4 | -2.331405197 |
| LAMB3 | -2.326909584 |
| COG8 | -2.3087782 |
| FARSB | -2.3087782 |
| PLEKHH2 | -2.3087782 |
| TNFRSF21 | -2.3087782 |
| RERE | -2.3087782 |
| SETD7 | -2.3087782 |
| 2010010A06RIK | -2.3087782 |
| ARAP1 | -2.3087782 |
| MTFP1 | -2.3087782 |
| CARD6 | -2.299393191 |
| GPCPD1 | -2.298441608 |
| ANXA3 | -2.291591463 |
| CCNT2 | -2.280049363 |
| ANKRD65 | -2.271257976 |
| CXCR6 | -2.260711964 |
| NAPB | -2.247377002 |
| EPB41L2 | -2.245719334 |
| SERPINB9B | -2.237093878 |
| SNRPE | -2.234784571 |
| TSSK2 | -2.227637527 |
| OSBP | -2.224590331 |
| KLHL15 | -2.221844546 |
| FBXO44 | -2.220298322 |
| KCTD9 | -2.218696302 |
| SNORD93 | -2.212779628 |
| FRAT2 | -2.189648147 |
| DHX35 | -2.189648147 |
| IDO2 | -2.189648147 |
| CPT1C | -2.189648147 |
| MIR7231 | -2.181622471 |
| BC031361 | -2.17925625 |
| RPS2 | -2.178510057 |
| S100A13 | -2.171454334 |
| GRSF1 | -2.166567042 |
| PRMT8 | -2.158545126 |
| PBXIP1 | -2.158545126 |
| LCTL | -2.156614782 |
| SELENBP1 | -2.151112289 |
| B9D1 | -2.144577498 |
| LOC101055843 | -2.132011726 |
| RELL1 | -2.122204582 |
| RIPK1 | -2.121007487 |
| SPG11 | -2.117102211 |
| TBX5 | -2.105426138 |
| ERO1LB | -2.105426138 |
| RAB10OS | -2.105426138 |
| BRI3 | -2.105426138 |
| ZFP963 | -2.105426138 |
| MIDN | -2.105426138 |
| ATP5MPL | -2.105426138 |
| GNG8 | -2.105426138 |
| FGF15 | -2.105426138 |
| OGG1 | -2.105426138 |
| MIR1953 | -2.105426138 |
| GM12530 | -2.100533229 |
| XPO1 | -2.097382332 |
| MAP6 | -2.091298801 |
| S100A8 | -2.091298801 |
| SLC38A6 | -2.078994949 |
| SLC22A21 | -2.078994949 |
| PYCR2 | -2.078994949 |
| GM9159 | -2.078994949 |
| ERP27 | -2.078994949 |
| FAM102A | -2.078300975 |
| IFT52 | -2.076071276 |
| GM9530 | -2.075365419 |
| AKR1A1 | -2.074867134 |
| 2010016I18RIK | -2.066637984 |
| KCTD2 | -2.066072865 |
| GM15816 | -2.054099703 |
| NAP1L5 | -2.049588216 |
| GGA3 | -2.047734974 |
| ARHGAP33 | -2.047734974 |
| PRELP | -2.044056834 |
| MYL9 | -2.035371069 |
| TGM1 | -2.027813044 |
| EGFR | -2.025034478 |
| MIR6951 | 2.017661821 |
| PON1 | 2.018327448 |
| TMEM108 | 2.018327448 |
| TMEM170B | 2.018327448 |
| WFDC11 | 2.023486487 |
| GM16386 | 2.023486487 |
| LIN37 | 2.023486487 |
| DMD | 2.034203978 |
| PAOX | 2.04021365 |
| MAGOH | 2.043412438 |
| GM35060 | 2.052564081 |
| DDO | 2.052564081 |
| FAM120AOS | 2.057602343 |
| GM5086 | 2.061458795 |
| ITGB7 | 2.062348393 |
| NDUFA13 | 2.06262307 |
| DDX20 | 2.066525375 |
| 4933425B07RIK | 2.069837518 |
| D130020L05RIK | 2.071874598 |
| POLR2D | 2.074362433 |
| UBE2U | 2.085511271 |
| 4930459C07RIK | 2.090246611 |
| BC030867 | 2.09583632 |
| ACTR1B | 2.102723035 |
| SPRN | 2.112160543 |
| ADIG | 2.113568384 |
| SDC4 | 2.113568384 |
| CCDC177 | 2.119493345 |
| JPT2 | 2.12350521 |
| SYNJ2BP | 2.12350521 |
| RBM47 | 2.12350521 |
| UBE2C | 2.128302121 |
| AK1 | 2.128302121 |
| MYO5B | 2.146034476 |
| POLDIP3 | 2.154498051 |
| DCBLD1 | 2.154564817 |
| MRO | 2.155361053 |
| 9530052E02RIK | 2.155361053 |
| 4933427D14RIK | 2.160053375 |
| OLFR663 | 2.160053375 |
| TBC1D4 | 2.164383933 |
| CD2 | 2.169478458 |
| ST8SIA2 | 2.179299137 |
| SATL1 | 2.195698486 |
| ZMYND10 | 2.2028465 |
| GM23363 | 2.204137198 |
| KLHL36 | 2.220003004 |
| WDR63 | 2.221533406 |
| CACUL1 | 2.234279423 |
| TTC1 | 2.236502594 |
| GM15880 | 2.255708894 |
| NDST2 | 2.255708894 |
| TPCN2 | 2.255708894 |
| BICDL1 | 2.262566369 |
| UBE2QL1 | 2.284803262 |
| TRPV2 | 2.284803262 |
| ABTB1 | 2.284803262 |
| LMO4 | 2.286234763 |
| ZFP846 | 2.302302106 |
| AMD2 | 2.337151242 |
| DIXDC1 | 2.341288828 |
| CDC26 | 2.34952857 |
| ESPNL | 2.352020325 |
| MOB3B | 2.35407291 |
| LARP1B | 2.372771501 |
| EBP | 2.372771501 |
| FNDC3B | 2.388841372 |
| RERG | 2.399619308 |
| ERICH6 | 2.415724017 |
| SEC11C | 2.422075227 |
| BACH2OS | 2.451860994 |
| PLXNC1 | 2.455682755 |
| ZMYM2 | 2.459494419 |
| RBM17 | 2.481044225 |
| CRISPLD2 | 2.518269591 |
| MIEF2 | 2.526839159 |
| RGS20 | 2.530467615 |
| ACP5 | 2.535893247 |
| INTS14 | 2.537941972 |
| SCOC | 2.542478974 |
| TMEM62 | 2.554558554 |
| SEC16B | 2.554558554 |
| MRAS | 2.554558554 |
| COX15 | 2.580589136 |
| ZFP939 | 2.585248483 |
| PGM1 | 2.601566162 |
| UNC93A | 2.605011679 |
| BCAP31 | 2.608448987 |
| ERN2 | 2.624507798 |
| ILRUN | 2.631282293 |
| SETX | 2.650428936 |
| NOL12 | 2.650682998 |
| FOS | 2.657083078 |
| RBCK1 | 2.672612413 |
| WASF2 | 2.675892553 |
| TFF2 | 2.675892553 |
| SIAE | 2.694459948 |
| 4930449I24RIK | 2.697690844 |
| SRSF2 | 2.699840761 |
| MZT2 | 2.735062854 |
| CLUH | 2.743453463 |
| CCT4 | 2.760114156 |
| CBLN4 | 2.764261637 |
| ABCD2 | 2.766445083 |
| PKP3 | 2.781732511 |
| ECHS1 | 2.787809 |
| CUX1 | 2.790837674 |
| ZFAND4 | 2.800720447 |
| EFCAB2 | 2.80799155 |
| GOLGA4 | 2.810179583 |
| DEPTOR | 2.831971255 |
| MSH2 | 2.910454912 |
| EMB | 2.913236992 |
| ANAPC13 | 2.923494257 |
| 3830432H09RIK | 2.926251329 |
| MIR103-2 | 2.927627891 |
| CYP24A1 | 2.932033013 |
| SIK1 | 2.962715975 |
| NAT8F2 | 2.968077384 |
| QRFP | 2.977018151 |
| MIR3092 | 2.983258555 |
| GALE | 3.00351273 |
| ARF1 | 3.020909274 |
| GM20544 | 3.044080791 |
| RNASEH2B | 3.055085411 |
| 4930413E15RIK | 3.112321827 |
| VPS45 | 3.126493837 |
| ATL1 | 3.153009156 |
| BAHD1 | 3.165047374 |
| 1700028P15RIK | 3.221533406 |
| PDLIM2 | 3.236502594 |
| MCRIP1 | 3.253515146 |
| SLC7A6 | 3.255708894 |
| MIR5103 | 3.255708894 |
| MCPH1 | 3.268157807 |
| CMTM3 | 3.268157807 |
| EPHB4 | 3.297004685 |
| HDDC2 | 3.301258854 |
| KRT23 | 3.331632687 |
| 1700016K05RIK | 3.396640278 |
| SLC2A4RG-PS | 3.470309558 |
| CHRNA4 | 3.585248483 |
| GNPNAT1 | 3.740323553 |
| CRTC1 | 3.983258555 |
| TOMM34 | 4.30518665 |
| ZC3H12C | 4.481703745 |
